# Data on free and bound volatile compounds in six *Ribes nigrum* L. blackcurrant cultivars

**DOI:** 10.1016/j.dib.2018.01.090

**Published:** 2018-02-05

**Authors:** Yaran Liu, Shaoyang Wang, Jie Ren, Guanshen Yuan, Yiqing Li, Bolin Zhang, Baoqing Zhu

**Affiliations:** Beijing Key Laboratory of Forestry Food Processing and Safety, Department of Food Science, College of Biological Sciences and Biotechnology, Beijing Forestry University, Beijing 100083, China

## Abstract

The data investigated 198 volatile compounds of six currant cultivars grown in China which is analyzed by SPME–GC–MS. Volatile compounds in these currant samples were identified by two methods, comparing retention indices with reference standards and matching mass spectrum in the NST11 library. A synthetic currant matrix prepared according to the currant juice condition were extracted and analyzed using the same extraction procedure as the currant samples. The standard curve was generated for quantification of volatile compounds. For the volatiles without the available standard, the data provided consulting standards that had the same carbon atom or the similar functional structure for quantification. Further interpretation and discussion can be seen in article entitled “Characterization of Free and Bound Volatile Compounds in Six *Ribes nigrum* L. Blackcurrant Cultivars” (Liu et al., 2018) [1].

**Specifications Table**

**Table:** 

Subject area	*Chemistry*
More specific subject area	*Aroma*
Type of data	*Table*
How data was acquired	*GC–MS (Agilent 7890 gas chromatography and an Agilent 5975 mass spectrometer) with automated HS-SPME*
Data format	*Analyzed*
Experimental factors	*Each currant cultivar was mashed and centrifuge to obtain currant juice. The currant juice was mixed with NaCl and 4-methyl-2-pentanol (internal standard). The bound volatile extraction was released by AR2000 enzyme solution.*
Experimental features	*The free and bound volatile extraction was analyzed by HS-SPME followed by GC–MS using a 60 m* × *0.25 mm, 0.25 µm thickness HP-INNOWAX capillary column.*
Data source location	*The horticultural experimental station at the Northeast Agricultural University in China (latitude 44°04′ and longitude 125*^*°*^*42′*
)Data accessibility	*Data is provided with this article.*

**Value of the data**

• This data provided physicochemical parameters of six currant cultivars for further studies of currant quality control.
• Total 198 Volatile compounds were identified in six currant cultivars by GC–MS.
• The standard curve of some volatile compounds was generated for quantification of volatile compounds by GC–MS. The data can be used for reference of volatiles quantification.
• The data calculated the retention indices of volatiles compounds that can be used for qualitative analysis by GC–MS.

## 1 Data

See Table 1, Table 2, Table 3.

**Table 1:** Physicochemical parameters of six currant cultivars.

Cultivar	Juice yield (%)	pH	Total soluble solid (°Brix)	Titratable acid^a^ (g/L)
Sofya	65.07 ± 1.73 b	2.66 ± 0.01 a	14.80 ± 0.14 a	3.34 ± 0.13 ab
Yadrionaya	66.36 ± 1.10 b	2.68 ± 0.01 ab	15.04 ± 0.00 b	3.15 ± 0.09 a
Liangye	53.06 ± 0.64 a	2.81 ± 0.01 c	16.15 ± 0.07 c	4.03 ± 0.04 bc
Risagar	52.49 ± 1.22 a	2.68 ± 0.03 ab	17.25 ± 0.07 d	4.52 ± 0.21 c
Fertodi	51.46 ± 2.06 a	2.65 ± 0.02 a	18.05 ± 0.21 f	4.25 ± 0.43 c
Brodtrop	60.45 ± 4.46 ab	2.74 ± 0.00 b	15.05 ± 0.21 ab	3.06 ± 0.04 a

a expressed as citric acid. Data are mean ± standard deviation of triplicate tests. Different letters in each column indicate significant differences at a significant level of 0.05.

**Table 2:** Standard information of compounds.

Compounds	CAS no.	Characteristic Ions (*m/z*)	Quantitative ion (*m/z*)	Range (μg L^-1^)		Regression equation	*R*^2^
				Max	Min		
***Alcohols***							
Heptanol	111706	70,564,355	70	211.00	0.21	*y* = 1147.1*x* + 1.4069	0.976
1-Octanol	111875	567,084	56	6.56	0.01	*y* = 70.259*x* + 0.1283	0.991
Isopentanol	123513	55,424,341	55	1413.80	0.69	*y* = 19,779*x* + 31.211	0.952
Isohexanol	626891	56,414,342	56	94.00	1.47	*y* = 5358.2*x* + 0.8578	0.990
2-Heptanol	543497	454,355	45	51.50	0.10	*y* = 748.37*x* + 0.2814	0.994
1-Hexanol	111273	56,434,155	56	493.50	0.48	*y* = 1546.3*x* – 5.0952	0.885
(*Z*)-3-Hexen-1-ol	928961	6,741,395,582	67	2035.00	3.97	*y* = 13,476*x* + 2.371	0.990
3-Octanol	589980	59,558,341	59	12.00	0.05	*y* = 238.79*x* + 0.2758	0.993
(*E*)-2-Hexene-1-ol	928950	57,418,227	57	103.13	25.78	*y* = 16,945*x* + 36.908	0.914
Benzylalcohol	100516	79,108,107	79	1135.00	17.73	*y* = 36,338*x* + 36.142	0.994
							
**Aldehyde**							
Decanal	112312	4,341,575,544	43	19.40	0.02	*y* = 2272.1*x* – 0.2855	0.962
Hexanal	66251	4,456,414,357	44	7870.00	3.84	*y* = 7081*x* – 8.15	0.988
(*E*)-2-Hexenal	6728263	41,423,983	83	40,220.00	4.91	*y* = 11,726*x* – 704.46	0.982
Nonanal	124196	57,414,356	57	13.00	3.25	*y* = 648.61*x* + 1.9932	0.884
Octanal	124130	4,344,415,684	43	6.56	0.01	*y* = 70.259*x* + 0.1283	0.991
Benzaldehyde	100527	771,061,055,150	77	58.75	0.46	*y* = 1782*x* + 1.4342	0.993
							
***Acids***							
Butyric Acid	107926	607,341	60	120.00	15.00	*y* = 21,865*x* + 42.766	0.907
Hexanoic acid	142621	6,073,414,387	60	104.38	26.09	*y* = 8519.9*x* + 30.542	0.781
Heptanoic acid	111148	607,343	60	580.00	9.06	*y* = 5021.9*x* + 40.935	0.952
Octanoic Acid	124072	6,073,434,155	60	2210.00	138.13	*y* = 35,255*x* + 219.26	0.933
							
***Esters***							
Ethyl acetate	141786	43	43	7440.00	116.25	*y* = 7923.88*x* + 0.0075	0.999
Ethyl 2-hydroxybenzoate	118616	120	120	31.50	0.25	*y* = 537.33*x* + 1.7861	0.963
Ethyl butanoate	105544	714,329	71	103.20	3.23	*y* = 1624.7*x* + 6.7119	0.976
Isoamyl acetate	123922	43,705,587	70	202.20	1.58	*y* = 948.91*x* + 9.6091	0.953
Ethyl hexanoate	123660	8899	88	193.60	0.38	*y* = 628.86*x* + 9.0529	0.921
Hexyl acetate	142927	435,684	84	50.30	0.20	*y* = 739.31*x* + 0.1564	0.985
Ethyl octanoate	111115	7487	74	3.00	0.38	*y* = 318.11*x* + 0.3035	0.992
Ethyl caprylate	106321	88,127	88	103.30	0.05	*y* = 1049.9*x* + 4.7153	0.945
							
***Terpenoids***							
Linalool	78706	71,414,393	71	0.15	0.00	*y* = 3.4965*x* + 0.0045	0.996
Neral	106263	41	41	0.01	0.04	*y* = 0.3723*x* + 0.0057	0.960
Paracymene	99876	11,913,491,120	119	22.00	0.17	*y* = 380.08*x* + 1.7322	0.926
Nerol	106252	69,419,368	69	0.02	0.00	*y* = 0.1065*x* + 0.0005	0.968
Geraniol	106241	69,41,6829	69	0.03	0.00	*y* = 0.1597*x* + 0.0027	0.966
Sulcatone	110930	43	43	120.00	0.12	*y* = 645.87*x* + 0.9362	0.993
β-Myrcene	123353	41,936,939	41	0.02	0.00	*y* = 1.2298*x* + 9E–05	0.958
Furan linalool oxide				0.02	0.00	*y* = 2.8454*x* – 6E–05	0.971
α-Terpineol	98555	5,993,121,136	59	0.11	0.00	*y* = 6.6321x + 0.0026	0.978
(*Z*)-β-Damascenone	23726934	121	121	2.27	0.28	*y* = 2223*x* – 0.0036	0.995
(*E*)-β-Damascenone	23726934	69	69	2.27	0.04	*y* = 76.932*x* – 0.056	0.994
							
***Benzene***							
Benzaldehyde	100527	771,061,055,150	77	58.75	0.46	*y* = 1782*×* + 1.4342	0.993
Methyl 4-hydroxybenzoate	119368	120,152	120	69.00	0.54	*y* = 378.52*×* + 6.2031	0.910
2-Phenylethanol	60128	91,122	91	649.38	5.07	*y* = 27,424*×* + 11.581	0.995
Phenol	108952	946,665	94	43.40	0.01	*y* = 5832.8*×* – 1.562	0.979
*p*-Ethylguaiacol	2785899	137, 152	137	39.60	0.62	*y* = 729.32*×* + 2.8065	0.960
*p*-Ethylphenol	123079	107,122	107	6.23	0.39	*y* = 5730*×* + 0.4765	0.976
Styrene	100425	104,103,787,751	104	129.00	1.01	*y* = 1228*×* + 5.2634	0.960

**Table 3:** The volatile compounds in six currant cultivars.

NO.	NO.^a^	Compound	RI	Quantitative analysis^b^	Quantification *m/z*^c^	Quantitative standard curve^d^	Classification
1	A1	Acetic acid	1449	RI, Mass	60	Butyric Acid	Acids
2	A2	Propionic Acid	1534	RI, Mass	74	Butyric Acid	Acids
3	A3	Isobutyric acid	1563	RI, Mass	43	Butyric Acid	Acids
4	A4	Pivalic acid	1573	RI, Mass	57	Ethyl caprylate	Acids
5	A5	Butyric Acid	1622	Str, RI, Mass	60	Butyric Acid	Acids
6	A6	Isovaleric acid	1664	RI, Mass	60	Butyric Acid	Acids
7	A7	2-Ethylbutanoic acid	1665	RI, Mass	74	Butyric Acid	Acids
8	A8	Valeric acid	1732	RI, Mass	60	Hexanoic acid	Acids
9	A9	Hexanoic acid	1840	Str, RI, Mass	60	Hexanoic acid	Acids
10	A10	2-Ethylhexanoic acid	1944	RI, Mass	73	Hexanoic acid	Acids
11	A11	Heptanoic acid	1947	Str, RI, Mass	60	Heptanoic acid	Acids
12	A12	Octanoic Acid	2052	Str, RI, Mass	60	Octanoic Acid	Acids
13	A13	Nonanoic acid	2155	RI, Mass	60	Octanoic Acid	Acids
14	L14	Hexanal	1041	Str, RI, Mass	56	Hexanal	Aldehyde
15	L15	(*E*)-2-Hexenal	1220	Str, RI, Mass	41	(*E*)-2-Hexenal	Aldehyde
16	L16	Octanal	1280	Str, RI, Mass	43	Octanal	Aldehyde
17	L17	(*E*)-2-Heptenal	1323	RI, Mass	41	Hexanal	Aldehyde
18	L18	Nonanal	1385	Str, RI, Mass	57	Nonanal	Aldehyde
19	L19	1-Formyl-5-ethylcyclopentene	1414	RI, Mass	124	Decanal	Aldehyde
20	L20	(*E*)-2-Octenal	1428	RI, Mass	41	Decanal	Aldehyde
21	L21	Decanal	1492	Str, RI, Mass	43	Decanal	Aldehyde
22	L22	Benzaldehyde	1523	Str, RI, Mass	77	Benzaldehyde	Aldehyde
23	–	*p*-Tolualdehyde	1650	RI, Mass	91	Benzaldehyde	Aldehyde
24	L24	2-Isopropenyl-5-methylhex-4-enal	1684	Mass	69	Decanal	Aldehyde
25	L25	(*E,E*)-2,4-Nonadienal	1703	RI, Mass	81	Decanal	Aldehyde
26	L26	Cuminaldehyde	1785	RI, Mass	133	Benzaldehyde	Aldehyde
27	L27	3,4-Dimethylbenzaldehyde	1818	RI, Mass	133	Benzaldehyde	Aldehyde
28	B28	Styrene	1232	Str, RI, Mass	104	Styrene	Benzene
29	B29	2,5-Dimethylstyrene	1429	RI, Mass	117	Benzylalcohol	Benzene
30	B30	*p*-Cymenene	1411	RI, Mass	117	Benzylalcohol	Benzene
31	B31	2,4-Dimethylstyrene	1415	RI, Mass	117	Benzylalcohol	Benzene
32	B32	2-Allyltoluene	1420	RI, Mass	117	Benzylalcohol	Benzene
33	B33	Hypnon	1651	RI, Mass	105	Benzaldehyde	Benzene
34	B34	Veratrol	1704	RI, Mass	138	*p*-Ethylguaiacol	Benzene
35	B35	3-Hydroxy-3-phenylbutan-2-one	1756	Mass	43	2-Phenylethanol	Benzene
36	B36	Methyl 4-hydroxybenzoate	1777	Str, RI, Mass	120	Methyl 4-hydroxybenzoate	Benzene
37	B37	2-Methylnaphthalene	1857	RI, Mass	142	Benzylalcohol	Benzene
38	B38	1-Methylnaphthalene	1891	RI, Mass	142	Benzylalcohol	Benzene
39	B39	Butylhydroxytoluene	1913	RI, Mass	205	Benzaldehyde	Benzene
40	B40	1,2-Benzisothiazole	1959	RI, Mass	135	Benzylalcohol	Benzene
41	B41	5-Hydroxyindan	2481	Mass	133	*p*-Ethylphenol	Benzene
42	B42	Guaiacol	1859	Str, RI, Mass	109	Guaiacol	Volatile Phenols
43	B43	*o*-Cresol	1998	RI, Mass	108	*p* -Ethylphenol	Volatile Phenols
44	B44	Phenol	2001	Str, RI, Mass	94	Phenol	Volatile Phenols
45	B45	*p*-Ethylguaiacol	2027	Str, RI, Mass	137	*p*-Ethylguaiacol	Volatile Phenols
46	B46	2,5-Xylenol	2072	RI, Mass	122	*p*-Ethylphenol	Volatile Phenols
47	B47	*p*-Cresol	2077	RI, Mass	107	*p* -Ethylphenol	Volatile Phenols
48	–	Eugenol	2160	RI, Mass	164	*p*-Ethylphenol	Volatile Phenols
49	B49	*p*-Ethylphenol	2165	Str, RI, Mass	107	*p*-Ethylphenol	Volatile Phenols
50	B50	4-Vinylguaiacol	2187	RI, Mass	150	*p*-Ethylguaiacol	Volatile Phenols
51	B51	2,4-Di-tert-butylphenol	2289	RI, Mass	191	*p*-Ethylphenol	Volatile Phenols
52	B52	4-Formyl-2,6-di-tert-butylphenol	2461	Mass	219	*p*-Ethylphenol	Volatile Phenols
53	E53	Isoamyl acetate	1114	Str, RI, Mass	43	Isoamyl acetate	Acetate Esters
54	E54	Hexyl acetate	1261	Str, RI, Mass	43	Hexyl acetate	Acetate Esters
55	E55	(*Z*)-3-Hexenol acetate	1308	RI, Mass	43	Ethyl hexanoate	Acetate Esters
56	E56	(*E*)-2-Hexenol acetate	1324	RI, Mass	43	Ethyl hexanoate	Acetate Esters
57	E57	Furfuryl acetate	1530	RI, Mass	43	Ethyl caprylate	Acetate Esters
58	E58	Bornyl acetate	1576	RI, Mass	95	Ethyl caprylate	Acetate Esters
59	E59	Benzyl acetate	1727	RI, Mass	108	Ethyl 2-hydroxybenzoate	Acetate Esters
60	E60	2-Phenethyl acetate	1816	RI, Mass	104	Ethyl 2-hydroxybenzoate	Acetate Esters
61	E61	Ethyl acetate	691	Str, RI, Mass	43	Ethyl acetate	Ethyl esters
62	E62	Ethyl butanoate	899	Str, RI, Mass	71	Ethyl butanoate	Ethyl esters
63	E63	Ethyl hexanoate	1222	Str, RI, Mass	88	Ethyl hexanoate	Ethyl esters
64	E64	Ethyl octanoate	1387	Str, RI, Mass	74	Ethyl octanoate	Ethyl esters
65	E65	Ethyl 2-hydroxybutanoate	1403	RI, Mass	59	Ethyl 2-hydroxybenzoate	Ethyl esters
66	E66	Ethyl caprylate	1425	Str, RI, Mass	88	Ethyl caprylate	Ethyl esters
67	E67	Ethyl 3-hydroxybutyrate	1516	RI, Mass	43	Ethyl 2-hydroxybenzoate	Ethyl esters
68	E68	Ethyl benzoate	1666	RI, Mass	105	Ethyl 2-hydroxybenzoate	Ethyl esters
69	E69	Ethyl 3-hydroxyhexanoate	1677	RI, Mass	71	Ethyl 2-hydroxybenzoate	Ethyl esters
70	E70	Ethyl 2-hydroxybenzoate	1813	Str, RI, Mass	120	Ethyl 2-hydroxybenzoate	Ethyl esters
71	E71	Methyl butanoate	785	RI, Mass	74	Ethyl butanoate	Other Esters
72	E72	Methyl caproate	1186	RI, Mass	74	Ethyl butanoate	Other Esters
73	E73	Butyl butanoate	1207	RI, Mass	71	Ethyl butanoate	Other Esters
74	E74	Methyl 2-hydroxybutyrate	1379	RI, Mass	59	Ethyl 2-hydroxybenzoate	Other Esters
75	E75	Methyl 3-hydroxybutanoate	1478	Mass	43	Ethyl 2-hydroxybenzoate	Other Esters
76	E76	Methyl 2-hydroxy-3-methylpentanoate	1490	RI, Mass	90	Ethyl 2-hydroxybenzoate	Other Esters
77	E77	Methyl 2-hydroxyhexanoate	1575	RI, Mass	69	Ethyl 2-hydroxybenzoate	Other Esters
78	E78	Methyl decanoate	1590	RI, Mass	74	Ethyl caprylate	Other Esters
79	E79	Methyl benzoate	1620	RI, Mass	105	Ethyl 2-hydroxybenzoate	Other Esters
80	E80	Methyl 3-hydroxycaproate	1645	RI, Mass	43	Ethyl 2-hydroxybenzoate	Other Esters
81	E81	Methyl 2-hydroxyoctanoate	1784	Mass	97	Ethyl 2-hydroxybenzoate	Other Esters
82	E82	3-Hydroxy-2,4,4-trimethylpentyl 2-methylpropanoate	1869	RI, Mass	71	Ethyl caprylate	Other Esters
83	E83	2,4,4-trimethyl-1,3-pentanediyl bis(2-methylpropanoate)	1880	Mass	71	Ethyl caprylate	Other Esters
84	E84	1-hydroxy-2,4,4-trimethyl-3-pentanyl 2-methylpropanoate	1885	Mass	71	Ethyl caprylate	Other Esters
85	E85	Methyl 5-methylsalicylate	1902	Mass	134	Ethyl 2-hydroxybenzoate	Other Esters
86	E86	Diisobutyl glutarate	2008	Mass	115	Ethyl caprylate	Other Esters
87	E87	2-Isobutoxyethyl benzoate	2128	Mass	105	Ethyl 2-hydroxybenzoate	Other Esters
88	H88	2-Pentanol	1135	RI, Mass	45	2-Heptanol	Higher Alcohols
89	H89	Isopentanol	1214	Str, RI, Mass	55	Isopentanol	Higher Alcohols
90	H90	Pentyl alcohol	1253	RI, Mass	42	1-Hexanol	Higher Alcohols
91	H91	3-Heptanol	1291	RI, Mass	59	2-Heptanol	Higher Alcohols
92	H92	2-Methyl-1-pentanol	1297	RI, Mass	43	Isopentanol	Higher Alcohols
93	H93	4-Pentenol	1299	RI, Mass	67	(*E*)-2-Hexene-1-ol	Higher Alcohols
94	H94	(*E*)-2-Penten-1-ol	1309	RI, Mass	57	(*E*)-2-Hexene-1-ol	Higher Alcohols
95	H95	Isohexanol	1310	Str, RI, Mass	56	Isohexanol	Higher Alcohols
96	H96	2-Heptanol	1315	Str, RI, Mass	45	2-Heptanol	Higher Alcohols
97	H97	3-Methylpentanol	1323	RI, Mass	56	Isohexanol	Higher Alcohols
98	H98	1-Hexanol	1349	Str, RI, Mass	56	1-Hexanol	Higher Alcohols
99	H99	4-Methyl-2-heptanol	1353	Mass	45	Isohexanol	Higher Alcohols
100	H100	6-Methyl-2-heptanol	1368	RI, Mass	45	Isohexanol	Higher Alcohols
101	H101	(*Z*)-3-Hexen-1-ol	1381	Str, RI, Mass	67	(*Z*)-3-Hexen-1-ol	Higher Alcohols
102	H102	4-Methyl-3-pentenol	1384	RI, Mass	41	Isohexanol	Higher Alcohols
103	H103	3-Octanol	1387	Str, RI, Mass	59	3-Octanol	Higher Alcohols
104	H104	(*E*)-2-Hexene-1-ol	1401	Str, RI, Mass	57	(*E*)-2-Hexene-1-ol	Higher Alcohols
105	H105	4-Methylhexanol	1424	RI, Mass	70	1-Octanol	Higher Alcohols
106	H106	Heptanol	1448	Str, RI, Mass	70	Heptanol	Higher Alcohols
107	H107	1-Octanol	1551	Str, RI, Mass	56	1-Octanol	Higher Alcohols
108	H108	Norbornyl alcohol	1561	Mass	94	1-Octanol	Higher Alcohols
109	H109	(*E*)-2-Octen-1-ol	1609	RI, Mass	57	(*E*)-2-Hexene-1-ol	Higher Alcohols
110	H110	(*Z*)-5-Octen-1-ol	1609	RI, Mass	67	(*E*)-2-Hexene-1-ol	Higher Alcohols
111	H111	1-Nonanol	1654	RI, Mass	56	1-Octanol	Higher Alcohols
112	H112	(*Z*)-3-Nonen-1-ol	1679	RI, Mass	81	(*E*)-2-Hexene-1-ol	Higher Alcohols
113	H113	1-Hydroxycumene	1756	RI, Mass	43	2-Phenylethanol	Higher Alcohols
114	H114	Decanol	1758	RI, Mass	70	1-Octanol	Higher Alcohols
115	H115	α-Phenylethanol	1810	RI, Mass	79	Benzylalcohol	Higher Alcohols
116	H116	*p*-Cymen-8-ol	1847	RI, Mass	43	2-Phenylethanol	Higher Alcohols
117	H117	Benzylalcohol	1875	Str, RI, Mass	79	Benzylalcohol	Higher Alcohols
118	H118	2-Phenylethanol	1911	Str, RI, Mass	91	2-Phenylethanol	Higher Alcohols
119	H119	2,4-Dimethylphenethyl alcohol	2017	Mass	119	2-Phenylethanol	Higher Alcohols
120	H120	*p*-Isopropylbenzyl alcohol	2098	RI, Mass	135	2-Phenylethanol	Higher Alcohols
121	K121	2-Octanone	1281	RI, Mass	43	Decanal	Ketones
122	K122	Acetoin	1297	RI, Mass	45	1-Hexanol	Ketones
123	K123	2,3-Octanedione	1315	RI, Mass	43	Decanal	Ketones
124	K124	Tyranton	1367	RI, Mass	43	Ethyl hexanoate	Ketones
125	K125	3-Methyl-3-cyclohexen-1-one	1431	Mass	67	Decanal	Ketones
126	K126	(*E,E*)-3,5-Octadien-2-one	1569	RI, Mass	95	Decanal	Ketones
127	K127	2-Furyl ethyl ketone	1569	RI, Mass	95	Styrene	Ketones
128	K128	Sabina ketone	1634	RI, Mass	81	Decanal	Ketones
129	K129	Cryptone	1674	RI, Mass	96	Styrene	Ketones
130	O130	Dimethyl trisulfide	1373	RI, Mass	126	Styrene	Others
131	O131	Tonkalide	1706	RI, Mass	85	Styrene	Others
132	O132	2-Methyl-2-butenolide	1720	RI, Mass	41	Styrene	Others
133	O133	5-Octanolide	1973	RI, Mass	99	Ethyl 3-hydroxybutanoate	Others
134	–	Coumaran	2361	RI, Mass	20	Benzyl alcohol	Others
135	T135	6-Methyl-5-hepten-2-ol	1456	RI, Mass	95	Ethyl caprylate	Norisoprenoids
136	–	Vinyldimethylcarbinol	934	RI, Mass	71	–	Terpenoids
137	T137	3-Karen	1127	RI, Mass	93	Terpinolen	Terpenoids
138	T138	Nopinen	1139	RI, Mass	93	Terpinolen	Terpenoids
139	T139	Menthadiene	1142	RI, Mass	93	Terpinolen	Terpenoids
140	T140	Terpilene	1156	RI, Mass	93	Terpinolen	Terpenoids
141	T141	β-Phellandrene	1187	RI, Mass	93	Terpinolen	Terpenoids
142	T142	Cineole	1199	RI, Mass	43	Terpinolen	Terpenoids
143	T143	Crithmene	1226	RI, Mass	93	Terpinolen	Terpenoids
144	T144	Paracymene	1253	Str, RI, Mass	119	Paracymene	Terpenoids
145	T145	Terpinolene	1265	RI, Mass	93	Terpinolen	Terpenoids
146	–	Prenol	1321	RI, Mass	71	–	Terpenoids
147	T147	Sulcatone	1333	Str, RI, Mass	43	Sulcatone	Terpenoids
148	T148	Rosoxide	1356	RI, Mass	139	Furan linalool oxide	Terpenoids
149	T149	3,4-Dimethyl-2,4,6-octatriene	1360	Mass	121	β-Myrcene	Terpenoids
150	T150	*cis*-Linaloloxide	1441	RI, Mass	59	Furan linalool oxide	Terpenoids
151	T151	Linalyl oxide A	1469	Mass	59	Furan linalool oxide	Terpenoids
152	T152	Camphor	1516	RI, Mass	95	α-Terpineol	Terpenoids
153	T153	Bornylene	1520	RI, Mass	121	Terpinolen	Terpenoids
154	T154	1,4-Dimethyl-4-acetcyclohexene	1521	RI, Mass	109	α-Terpineol	Terpenoids
155	T155	Linalool	1541	Str, RI, Mass	71	Linalool	Terpenoids
156	T156	L-4-terpineol	1591	RI, Mass	71	α-Terpineol	Terpenoids
157	T157	Caryophyllene	1592	RI, Mass	93	Terpinolen	Terpenoids
158	T158	*p*-Menth-1-en-9-al	1614	RI, Mass	94	α-Terpineol	Terpenoids
159	T159	Menthol	1637	RI, Mass	71	α-Terpineol	Terpenoids
160	T160	Pinocarveol	1653	RI, Mass	92	α-Terpineol	Terpenoids
161	T161	Cephreine	1656	RI, Mass	43	Linalool	Terpenoids
162	T162	2,2,3-Trimethyl-3-cyclopentene-1-ethanol	1658	Mass	139	α-Terpineol	Terpenoids
163	T163	Cephreine	1660	RI, Mass	43	Linalool	Terpenoids
164	T164	Borneol	1698	RI, Mass	95	α-Terpineol	Terpenoids
165	T165	Phellandral	1704	RI, Mass	109	Neral	Terpenoids
166	T166	6-(2-Hydroxy-2-propanyl)-3-methyl-2-cyclohexen-1-yl acetate	1715	Mass	59	α-Terpineol	Terpenoids
167	T167	6-Methyl-2-vinyl-5-hepten-1-ol	1726	Mass	41	Nerol	Terpenoids
168	T168	2-Hydroxycineol	1726	Mass	108	Furan linalool oxide	Terpenoids
169	T169	Lilac alcohol formate A	1731	Mass	55	Furan linalool oxide	Terpenoids
170	T170	Piperitone	1731	RI, Mass	82	α-Terpineol	Terpenoids
171	T171	Neral	1733	Str, RI, Mass	41	Neral	Terpenoids
172	T172	Carvone	1736	RI, Mass	82	α-Terpineol	Terpenoids
173	T173	Linalool oxide	1736	RI, Mass	68	Furan linalool oxide	Terpenoids
174	T174	Lilac alcohol B	1743	RI, Mass	55	Furan linalool oxide	Terpenoids
175	T175	*cis*-*p*-Menth-2-en-7-ol	1753	Mass	93	α-Terpineol	Terpenoids
176	T176	L-Verbenone	1754	Mass	107	Terpinolen	Terpenoids
177	T177	(*Z*)-β-Damascenone	1760	Str, RI, Mass	121	(*Z*)-β-Damascenone	Terpenoids
178	T178	D-Citronellol	1761	Mass	41	Nerol	Terpenoids
179	T179	1-(2,6,6-trimethyl-1,3-cyclohexadien-1-yl)-2-buten-1-one	1744	Mass	121	β-Ionone	Terpenoids
180	T180	*trans-p*-menth-2-en-7-ol	1772	Mass	93	α-Terpineol	Terpenoids
181	T181	1,7,7-Trimethylbicyclo [2.2.1] hept-5-en-2-ol	1776	Mass	108	α-Terpineol	Terpenoids
182	T182	7-Methyl-3-methylene-6-octen-1-ol	1782	RI, Mass	69	Geraniol	Terpenoids
183	T183	Lilac alcohol C	1786	RI, Mass	55	Furan linalool oxide	Terpenoids
184	T184	Perillal	1789	RI, Mass	68	α-Terpineol	Terpenoids
185	T185	Nerol	1796	Str, RI, Mass	69	Nerol	Terpenoids
186	T186	Isogeraniol	1808	RI, Mass	41	Nerol	Terpenoids
187	T187	(*E*)-β-Damascenone	1822	Str, RI, Mass	69	(*E*)-β-Damascenone	Terpenoids
188	T188	Lilac alcohol D	1827	RI, Mass	55	Furan linalool oxide	Terpenoids
189	T189	(*Z*)-Carveol	1833	RI, Mass	109	α-Terpineol	Terpenoids
190	T190	Geraniol	1843	Mass	69	Geraniol	Terpenoids
191	T191	*exo*-2-Hydroxycineole	1858	RI, Mass	108	Furan linalool oxide	Terpenoids
192	T192	*p*-Menth-1-en-9-ol	1937	RI, Mass	94	α-Terpineol	Terpenoids
193	T193	*p*-Mentha-1,4-dien-7-ol	1946	RI, Mass	79	α-Terpineol	Terpenoids
194	T194	Perilla alcohol	1993	RI, Mass	68	α-Terpineol	Terpenoids
195	T195	*p*-Menthadien-7-ol	2087	Mass	79	α-Terpineol	Terpenoids
196	T196	Spathulenol	2123	Mass	43	α-Terpineol	Terpenoids
197	T197	7-epi-α-selinene	2166	Mass	161	Terpinolene	Terpenoids
198	T198	Bisabolol	2207	RI, Mass	43	α-Terpineol	Terpenoids

a Numbers of compounds used for principal component analysis in article entitled “Characterization of Free and Bound Volatile Compounds in Six *Ribes nigrum* L. Blackcurrant Cultivars” [1].

b Quantitative Analysis – Str: identified with standard substance; RI: RI agreed with database of NIST11; Mass: mass spectrum agreed with the mass spectral database.

c Quantification *m/z* – the main fragment of compound for quantification.

d Quantitative Standard Curve – the compound was quantified by the standard curve of compounds with similar structure.

## 2 Experimental design, materials and methods

### 2.1 Currant samples

Six cultivars of currant (“Risagar”, “Fertodi”, “Brodtrop”, “Sofya”, “Yadrionaya”, and “Liangye”) were obtained from the horticultural experimental station at the Northeast Agricultural University in China (latitude 44°04′ and longitude 125°42′) at their maturity stage. The currant samples were frozen at -20°C prior to further analysis (Table 1).

### 2.2 Chemicals and reagents

Glucose, sodium hydroxide, sodium chloride, citric acid, and sodium dihydrogen phosphate were obtained from Beijing Chemical Works (Beijing, China). HPLC grade dichloromethane, ethanol, and methanol were purchased from Honeywell (Morris Township, NJ, USA). Pure water was obtained from Milli-Q purification system (Millipore, Bedford, MA, USA). The volatile standards used for identification were purchased from Sigma-Aldrich (St. Louis, MO, USA) with a purity above 98%. Other reagents were of analytical grade unless specifically noted.

### 2.3 Volatile extraction

Each currant cultivar was mashed and centrifuged to yield currant juice. The currant juice (5 mL) was mixed with NaCl (1.00 g) and 1.0018 g/L 4-methyl-2-pentanol (10 µL, internal standard) in a 15-mL glass vial containing a magnetic stirrer with a PTFE-silicon septum. The mixture was equilibrated on a heating and agitation platform at 40 °C for 30 min. The free volatile compounds in the sample were concentrated with headspace SPME regarding our previous report [2]. Each currant cultivar was conducted in three independent extractions.

The bound volatiles were released using AR2000 enzyme solution according to our published methods [2]. Afterwards, the bound volatile compounds were extracted and analyzed using the same SPME as the free volatile compounds.

### 2.4 GC–MS analysis

The volatile compounds analysis on GC followed our previous method [3]. An Agilent 7890 gas chromatography equipped with an Agilent 5975 mass spectrometer (Agilent Technologies, Santa Clara, CA, USA) was used to analyze volatile compounds. A 60 m × 0.25 mm, 0.25 µm thickness HP-INNOWAX capillary column (J&W Scientific, Folsom, CA, USA) was used to separate the volatile compounds using the carrier gas (helium) at 1 mL/min flow rate. The over temperature was programmed as follows: 50 °C held for 1 min, then increased from 50 °C to 220 °C at a rate of 3 °C /min and held at 220 °C for 5 min, and then increased to 250 °C at 5 °C /min and held at 250 °C for 5 min. The temperature of MS transfer line was set at 280 ^o^C. Mass spectrum was recorded at 70 eV with 130 °C in the electron impact (EI) mode. All scan mass from *m/z* 25 to 300 was recorded. C_6_-C_24_ alkane series (Supelco, Bellefonte, PA, USA) was analyzed under the same chromatographic conditions for calculation of retention indices of volatiles. The volatiles in currant were identified by comparing their retention indices and mass spectrum with reference standard. The volatiles without the available standard were tentatively identified by comparing their retention indices and mass spectrum with the NIST11 library (Table 3).

### 2.5 Standards analysis

A synthetic currant matrix was prepared regarding the physicochemical index of the currant juice. The synthetic currant matrix consisted of 170 g/L sugar and 3.5 g/L citric acid with its pH adjusted 4.0 using 5 M NaOH solution. Each external volatile standard was dissolved in HPLC-grade ethanol to generate the stock standard solution. These stock standard solutions were then combined using the synthetic matrix to form standard working solution. Afterwards, the standards working solution was diluted using the synthetic matrix to 18 successive levels. The standards were analyzed using the same extraction procedure as the currant samples, and analyzed under the same GC method. The standard curve was integrated using the peak area ratio of external volatile standard to internal standard versus the concentration of external standard (Table 2).
