# Supplementary material for: Data on free and bound volatile compounds in six Ribes nigrum L. blackcurrant cultivars
Source: Data Brief. 2018 Feb 5;17:926–37. doi: 10.1016/j.dib.2018.01.090 (PMC5988502; doi:10.1016/j.dib.2018.01.090)
Supplement: Supplementary file 1 — Supplementary material [file mmc1.docx]

Conflict of Interest

The authors declared that they have no conflicts of interest to this work.
